# A microRNA/Runx1/Runx2 network regulates prostate tumor progression from onset to adenocarcinoma in TRAMP mice

**DOI:** 10.18632/oncotarget.11992

**Published:** 2016-09-13

**Authors:** Nicholas H. Farina, Areg Zingiryan, Jacqueline A. Akech, Cody J. Callahan, Huimin Lu, Janet L. Stein, Lucia R. Languino, Gary S. Stein, Jane B. Lian

**Affiliations:** ^1^ Department of Biochemistry and University of Vermont Cancer Center, University of Vermont College of Medicine, Burlington, VT 05405, USA; ^2^ Department of Cell and Developmental Biology, University of Massachusetts Medical School, Worcester, MA 01655, USA; ^3^ Prostate Cancer Discovery and Development Program, Department of Cancer Biology, Sidney Kimmel Cancer Center, Thomas Jefferson University, Philadelphia, PA 19107, USA

**Keywords:** prostate cancer progression, AR, PTEN, TRAMP, miRNA targeting Runx

## Abstract

While decades of research have identified molecular pathways inducing and promoting stages of prostate cancer malignancy, studies addressing dynamic changes of cancer-related regulatory factors in a prostate tumor progression model are limited. Using the TRAMP mouse model of human prostate cancer, we address mechanisms of deregulation for the cancer-associated transcription factors, Runx1 and Runx2 by identifying microRNAs with reciprocal expression changes at six time points during 33 weeks of tumorigenesis. We molecularly define transition stages from PIN lesions to hyperplasia/neoplasia and progression to adenocarcinoma by temporal changes in expression of human prostate cancer markers, including the androgen receptor and tumor suppressors, Nkx3.1 and PTEN. Concomitant activation of PTEN, AR, and Runx factors occurs at early stages. At late stages, PTEN and AR are downregulated, while Runx1 and Runx2 remain elevated. Loss of Runx-targeting microRNAs, miR-23b-5p, miR-139-5p, miR-205-5p, miR-221-3p, miR-375-3p, miR-382-5p, and miR-384-5p, contribute to aberrant Runx expression in prostate tumors. Our studies reveal a Runx/miRNA interaction axis centered on PTEN-PI3K-AKT signaling. This regulatory network translates to mechanistic understanding of prostate tumorigenesis that can be developed for diagnosis and directed therapy.

## INTRODUCTION

Prostate cancer (PCa) is the most diagnosed cancer among men in the United States and is the second leading cause of cancer-related deaths [1]. Numerous tumor-derived human prostate cancer cell lines have elucidated mechanisms contributing to PCa cell phenotypes including the deregulation of oncogenes, tumor suppressors, and growth factors. Transcriptional regulators that activate biological pathways, such as the epithelial to mesenchymal transition (EMT), are well characterized to promote prostate tumorigenesis [2]. However, there still remains a gap in knowledge as to the mechanisms driving clinically relevant disease. The use of animal models that develop endogenous prostate tumors, such as the TRansgenic Adenocarcinoma of the Mouse Prostate (TRAMP) [3, 4], can add to our understanding of the mechanisms underlying PCa progression. Here, we report for the first time in TRAMP mice, dynamic expression changes of the transcription factors, Runx1 and Runx2, and microRNAs that contribute to deregulated expression of Runx proteins, during the transition of normal prostate tissue to adenocarcinoma.

Our laboratory, and others, have identified expression and functional activities of the Runx transcription factors in various cancers [5]. There is mounting evidence that Runx factors are transcriptional drivers of tumor progression [6]. Runx1 and Runx2 are expressed in the normal epithelium of exocrine glands [7], sites of origination for carcinomas. Both mutations [8, 9] and increased expression [10] of Runx1, a required factor for hematopoiesis, are associated with breast [11] and lung [12] cancer tumors. Runx2, an essential “master regulator” for bone formation, is abnormally expressed in aggressive breast and prostate cancer and activates genes associated with tumorigenesis and metastasis [11, 13, 14]. In data generated by the TCGA Research Network: http://cancergenome.nih.gov/ and analyzed with cBioPortal [15–17], mutations in Runx1 and Runx2 occur in 6.1% and 2.8%, respectively, of invasive breast carcinoma and, while these mutation rates are near 0% for prostate adenocarcinoma, the mRNA levels of Runx1 and Runx2 increase 4.8% and 3.6%, respectively, in men with PCa. Further, men with castration resistant PCa and androgen-independent mouse prostate tumors upregulate Runx1 [18]. Further, Runx2 is reported to be expressed in nearly 50% of patients with primary prostate tumors or PCa-induced bone metastases [19]. In addition, Runx1 and Runx2 are expressed in both early and late stage PCa-derived cell lines and promote expression of the prostate specific antigen (PSA) [20]. Abnormally high levels of Runx2, and their targeted genes, in human prostate tissue and cells lines positively correlate with tumor stage and aggressiveness [19, 21]. Our previous studies, and those of other laboratories, have documented the osteomimetic properties of Runx2 in primary tumors and that inhibition of Runx2 in breast and prostate cancer cells blocks metastasis and osteolytic bone disease mediated by human tumor cells in orthotopic mouse models [19, 21–23].

MicroRNAs (miRNA) have emerged as important biomarkers for nearly all cancers with hundreds of discovered direct mRNA/miRNA interactions elucidating their functional activity in silencing protein expression of individual genes [11, 24]. Furthermore, miRNAs that directly target Runx2, but are missing in aggressive breast cancer cells, have been shown to be effective in disease intervention by a miRNA replacement approach *in vivo* [25]. Moreover, direct evidence implicates miRNAs in the development of prostatic tumors and progression through adenocarcinoma [26, 27]. Among the most well studied miRNAs that promote carcinogenesis, miR-21 is upregulated in more aggressive prostate tumors [28] and directly targets tumor suppressors resulting in activation of tumorigenic pathways [29–32]. On the contrary, miR-203-3p is progressively downregulated in primary prostate tumors, is absent or lowly expressed in bone metastatic PCa cells and metastases, and suppresses Runx2 and other genes that promote PCa metastasis to bone [33]. Thus, miRNAs that target Runx1 or Runx2 and are deregulated during prostate tumorigenesis present exciting candidates for novel therapeutic intervention in men diagnosed with prostate cancer.

In the present studies, compelling questions related to temporal expression changes and functional significance of Runx1, Runx2, and Runx-targeting miRNAs were addressed in a true local progression model of PCa. The TRAMP mouse is ideal to study the dynamic expression of Runx1 and Runx2 in the transition from a normal gland to tumorigenic tissue as TRAMP prostates develop morphologically and histologically similar tumors to human prostate cancer [3, 4]. Common features of the TRAMP mouse and human prostate cancer include formation of prostatic intraepithelial neoplasia (PIN) lesions, transition to hyperplasia/neoplasia, progression to an adenocarcinoma and oftentimes mixed neuroendocrine disease, invasion of the surrounding tissue, and metastasis, most commonly to lung and peripheral lymphatic tissue [34–37]. To date, the TRAMP mouse has been widely used for validating dysregulated genes and their associated pathways *in vivo* and for testing novel inhibitors of prostate cancer progression [36]. However, previous studies primarily focused on histologic protein analyses of murine prostate tissue. Notably, a limited number of genome-wide gene expression studies are available and only end stage tumors in aged TRAMP animals with palpable and poorly differentiated tumors were examined [38, 39].

Here, we examine expression levels of known markers for early and advanced stages during prostate tumor progression in the TRAMP mouse to establish stages representing human PCa. In this context, we tested the hypothesis that aberrant Runx1 and Runx2 expression involves a mechanism where small, non-coding microRNAs deregulate Runx levels resulting in increased downstream signaling to drive tumorigenesis. We further postulate that those genes regulated by Runx1, Runx2, and miRNAs targeting Runx form an interaction network that promotes PCa progression and may reveal distinct and/or cooperative mechanisms (by Runx1/2) contributing to PCa. We find the androgen receptor and tumor suppressors, Nkx3.1 and PTEN, together define a transition from early to late stage disease in the TRAMP mouse. Runx1 and Runx2 continuously increase while miRNAs with predicted or validated repression of Runx factors decrease during tumor progression. In addition, we identify PTEN-PI3K-AKT signaling as mediated by a Runx/miRNA interaction axis.

## RESULTS

### Gene expression changes indicate two phases of local prostate tumorigenesis

**Figure 1:**
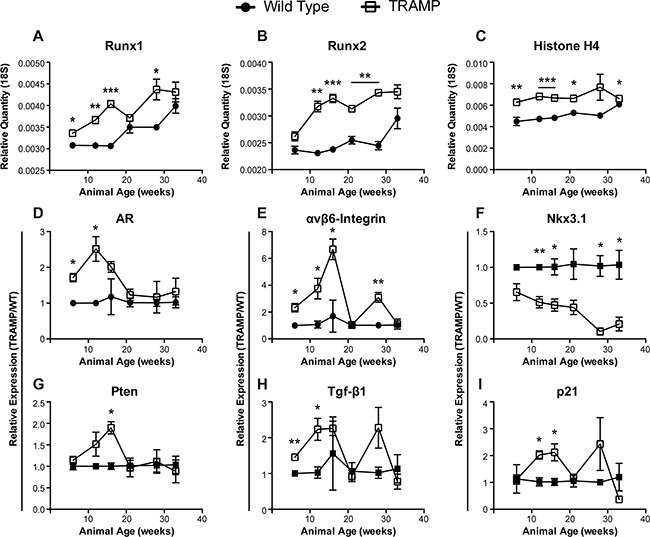
Gene expression changes in TRAMP prostate through disease progression qPCR levels of Runx1 (**A**), Runx2 (**B**), Histone H4 (**C**), androgen receptor (AR) (**D**), αvβ6-Integrin (**E**), Nkx3.1 (**F**), Pten (**G**), Tgf-β1 (**H**), and p21 (**I**) message in the ventral and lateral prostate lobes isolated from wild type (•) or TRAMP (□) animals. Animals were sacrificed at 6, 12, 16, 21, 28, and 33 weeks to follow tumor development. Both Runx1 (**A**) and Runx2 (**B**) transcriptional drivers of tumor progression are elevated in TRAMP prostates irrespective of animal age and increase throughout the time course. (**A-C**) Relative quantity calculated as 1/(2^(Ct,gene – Ct,18S)). (**D-I**) Relative expression calculated using the delta delta Ct method, 2^((Ct,gene – Ct,18S)_wild type_ – (Ct,gene – Ct,18S)_TRAMP_), at each time point. All expression values normalized to 18S. N = 3 animals per time per genotype. Error bars are SEM. * p < 0.05, ** p < 0.01, *** p < 0.001 compared to wild type.

We isolated RNA from transgenic and non-transgenic TRAMP mouse prostates spanning from adolescence, prior to histologic detection, through development of PIN lesions, hyperplasia, and adenocarcinoma (Supplementary Figure S1) [4]. Expression of Runx1 and Runx2 mRNA were assessed by qPCR at six time points in wild type and transgenic prostate lobes. By 12 weeks, when PIN lesions begin to appear (Supplementary Figure S1), Runx1 and Runx2 are significantly upregulated in the TRAMP prostate compared to wild type animals and remain at elevated levels through 33 weeks (Figure 1A, 1B). Histone H4, a marker of proliferation, while slightly elevated in TRAMP animals, remains constitutive through the time course in both wild type and transgenic mice, suggesting that the persistent increases of Runx1 and Runx2 in TRAMP prostates are not related to proliferative differences and likely result from prostatic tumorigenesis (Figure 1C).

As human prostate cancer advances, becoming more aggressive, response to hormone therapy is abrogated and the disease transitions to androgen independence, designated castration-resistant prostate cancer, leading to more invasive treatments and dramatically reduced quality of life [40]. The androgen receptor (AR) is more highly expressed in TRAMP prostates through initial disease development but is reduced by 21 weeks, remaining at similar levels in TRAMP and wild type prostates through 33 weeks (Figure 1D). This pattern of decreased AR in TRAMP mice may represent a transformation of the tumor to androgen independent disease. Thus, the TRAMP model provides an *in vivo* system for future study to elucidate molecular mechanisms involved in the transition to castration-resistant prostate cancer. AR has been directly tied to the expression of integrins in prostate cancer [41]. In addition, recent evidence implicates αvβ6-integrin in PCa progression, specifically in promoting an osteolytic program in metastatic tumor cells [21]. GEM models of prostate cancer do not develop metastatic bone lesions. Nonetheless, αvβ6-integrin is initially upregulated more than 6-fold in TRAMP prostates, as compared to non-transgenic mice, through the first 16 weeks, suggesting a role in disease onset and tumor development. Yet, by 21 weeks, levels are reduced to that of wild type mice (Figure 1E). In fact, this dip in expression is shared amongst other genes, including Runx1 and Runx2, and may indicate a dramatic shift in disease state occurring in TRAMP mice between 16 and 21 weeks (Figure 1).

To determine if the changes observed in Runx1, Runx2, AR, and αvβ6-integrin correspond to PCa biomarkers indicative of disease stage, we assayed for Nkx3.1, Pten, Tgf-β1, and p21. The tumor suppressors Nkx3.1 and Pten play an important and integrative role in prostate cancer development and progression to metastatic bone disease. Nkx3.1 negatively regulates epithelial cell propagation and thus, down regulation of Nkx3.1 results in increased cellular proliferation leading to neoplasia (PIN lesions) and hyperplasia [42]. In striking contrast to wild type tissue, Nkx3.1 decreases continuously in TRAMP prostates, is repressed more than 2-fold through 21 weeks and nearly 15-fold by 28 weeks (Figure 1F). While levels of Pten mRNA increase almost 2-fold by week 16, its expression rapidly decreases by week 21 and remain low through the time course (Figure 1G). Loss of Pten is associated with the progression from a localized prostate carcinoma to invasive and metastatic adenocarcinoma [43].

Both Tgf-β1 and p21 have described dual roles as tumor promoters and suppressors in cancer [reviewed in 44, 45]. Transgenic animals exhibit increasing levels of these factors by at least 2-fold up to 16 weeks with a sharp decline at week 21 (Figure 1H, 1I). Tgf-β1 and p21, as well as αvβ6-integrin, spike again at 28 weeks, which may reflect a sub-population or new foci of tumor cells that recapitulates the upregulation observed at tumor onset. The increases in Tgf-β1 and p21 levels at the indicated time points are likely to be associated with oncogenic activity. In contrast, the return to wild type levels at 21 and 33 weeks may reflect functions tied to metastatic castration-resistant prostate cancer rather than the driver role of Tgf- β in early tumorigenesis. Consistent with this interpretation is that loss of Tgf- β leads to an increase in metastases in PTEN null mice as well as development of invasive prostate cancer in mice with constitutive AKT signaling [46]. Furthermore, p21 directly regulates PI3K/AKT activation and is AR responsive [47] suggesting that the low levels at 21 weeks is also contributing to a transition to androgen insensitive disease, as indicated above for the decrease in AR by 21 weeks. In conclusion, we observe elevated Runx1 and Runx2 levels throughout disease progression that is associated with dynamic expression of PCa-associated genes (AR, αvβ6-integrin, Nkx3.1, Pten, Tgf-β1, and p21) that mark stages of prostate tumor development in the TRAMP mouse consistent with human disease.

### Loss of Runx1 and Runx2 inhibits prostate cell motility

**Figure 2:**
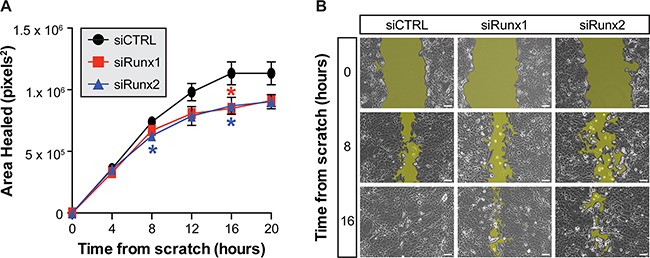
Inhibition of Runx1 and Runx2 reduces motility of TRAMP prostate cells The average area healed following a scratch wound in a monolayer of TRAMP-C2 cells is reduced following siRNA inhibition of Runx1 (red) or Runx2 (blue) as compared to non-targeting control (black) (**A**) Cells transfected with non-targeting control migrate to heal the wound by 16 hours while cells treated with siRunx1 or siRunx2 do not completely close the scratch (**B**) The threshold used to measure wound area is illustrated by the yellow highlights in each representative image. Three locations imaged per condition in three independent experiments from sequential cell passages. Error bars are SEM. * p < 0.05 compared to non-targeting control. Scale bar = 100μm.

The persistent elevation of Runx1 and Runx2 transcription factors in TRAMP prostate tissue throughout progression to adenocarcinoma suggests a pro-tumorigenic role in prostate cancer. Using the TRAMP-C2 cell line, which provides an *in vitro* model to elucidate molecular mechanisms of prostate cancer, we assessed the functional activities of Runx1 and Runx2 in PCa. Transfection of siRNAs against Runx1 and Runx2 in TRAMP-C2 cells reduces the area healed in response to a scratch wound made in the cell monolayer. Notably, inhibition of both Runx1 and Runx2 led to a 20% decrease in area healed by 12 hours as compared to non-targeting control, well before 100% closure of scratch wound (Figure 2). All scratch wounds healed by 20 hours. This result would be expected given the similarity between the DNA-binding domain of Runx1 and Runx2 and their potential to regulate similar gene cohorts. In addition, inhibition of Runx1 in an *in vitro* breast cancer model reduces cell migration [22]. Inhibition of Runx2 by miRNAs that result in decreased PTK2 and ROCK1 signaling, contribute to cell motility [25], and another study directly identified by ChIP-Seq Runx2 binding to cell adhesion and motility-related genes [48]. These data, together with the temporal increase in expression of Runx1 and Runx2 during prostate tumor growth (Figure 1A, 1B), is indicative that Runx factors contribute to prostate cancer development and progression.

### MicroRNAs targeting Runx1 and Runx2 decrease in the TRAMP prostate

A question remains as to how abnormal levels of Runx factors are modulated during prostate cancer progression. The mechanism leading to an increase in Runx expression during prostate tumorigenesis may occur at the transcriptional or post-transcriptional level. As miRNAs directly control Runx expression [49, 50] and are established epigenetic regulators of cancer initiation, tumor progression, and metastasis, we postulate that miRNAs targeting Runx1 and/or Runx2 would have reciprocal expression in TRAMP prostates. Well over 50 individual miRNAs are computationally predicted to target Runx1 and/or Runx2 (TargetScan v6.2). Of these miRNAs, 28 candidates were selected for further interrogation in wild type and transgenic TRAMP prostates, representing the spectrum from novel to established PCa-association and repression of Runx1 and/or Runx2. To identify deregulated miRNAs targeting Runx in prostate cancer development and early tumorigenesis, miRNA expression levels were determined at 6, 12, 16, and 21 weeks by qPCR. Most miRNAs that target either or both Runx1 and Runx2 decrease within this timeframe with ~25% of the candidates found to be significantly downregulated, reciprocal to increased levels of the Runx factors (Table 1). Of note, several miRNAs reported to regulate Runx1 and/or Runx2, such as miR-135a [50] and miR-203 [33], and are associated with invasive and metastatic PCa trend downwards but do not achieve significant differences between transgenic and non-transgenic animals. We conclude that these miRNAs likely contribute to advanced disease and distal metastasis, which are outside the scope of the TRAMP animal model.

**Table 1:** Expression of candidate miRNAs predicted to target Runx1 and Runx2 in TRAMP prostate over time

miRNA	Target	*Fold Difference*			
		6wk	12wk	16wk	21wk
mmu-miR-20a-5p	Runx1	1.15	1.63	1.65^*^	−1.05
mmu-miR-20b-5p	Runx1	1.00	1.44	1.48^*^	1.12
***mmu-miR-23b-5p***	***Runx1/Runx2***	-1.22^*^	***-1.28***	-1.61^*^	***-2.17***
mmu-miR-27a-3p	Runx1	−1.28	−1.10	−1.09	−1.45
mmu-miR-27b-3p	Runx1	−1.06	−1.02	−1.25^**^	−1.69
mmu-miR-30a-5p	Runx1/Runx2	−1.19	−1.18	−1.01	−2.08
mmu-miR-30c-5p	Runx1/Runx2	1.04	−1.09	−1.30	−2.13
mmu-miR-30e-5p	Runx1/Runx2	−1.04	−1.08	1.04	−1.27
mmu-miR-93-5p	Runx1	1.51^*^	2.08	2.27^*^	1.73^*^
mmu-miR-135a-5p	Runx1/Runx2	−1.12	−1.39	−1.05	−1.56
mmu-miR-135b-5p	Runx1/Runx2	−1.22	−1.67	−1.32	−1.39
***mmu-miR-139-5p***	***Runx1***	***-1.11***	***-1.67***	-2.08^*^	***-1.75***
mmu-miR-141-3p	Runx1	−1.28	1.30	2.33	−1.49
mmu-miR-181a-5p	Runx1	−1.04	−1.22	−1.33	−1.11
mmu-miR-200a-3p	Runx1	1.19	1.60	1.77^**^	1.46
mmu-miR-200b-3p	Runx1	1.22	1.32	1.07	−1.70
mmu-miR-203-3p	Runx2	1.12	−1.14	−1.18	−1.07
mmu-miR-204-5p	Runx2	1.27	1.56	1.28	−1.61
***mmu-miR-205-5p***	***Runx1/Runx2***	-1.47*	-2.64^**^	-2.94^**^	-4.35^*^
mmu-miR-214-3p	Runx1	−1.11	−1.48	−1.70	−1.76
mmu-miR-218-5p	Runx2	−1.22	−1.30	−1.16	−1.72
***mmu-miR-221-3p***	***Runx2***	***-1.20***	***-1.45***	-1.56^*^	-2.17^*^
mmu-miR-320-3p	Runx1/Runx2	−1.23^*^	−1.22	−1.67	−1.82
mmu-miR-324-5p	Runx2	−1.19	−1.09	−1.27	−1.49
mmu-miR-340-5p	Runx1/Runx2	1.38	2.39	2.53^**^	2.06
***mmu-miR-375-3p***	***Runx1/Runx2***	-1.27^***^	***-1.96***	-3.03*	-6.67^**^
***mmu-miR-382-5p***	***Runx1***	***-1.33***	***-1.28***	-2.50^**^	-2.22*
***mmu-miR-384-5p***	***Runx1/Runx2***	***1.15***	***1.15***	-2.38*	-2.94*

The levels of 28 selected miRNAs predicted to target Runx1, Runx2, or both (TargetScan v6.2) were determined by qPCR. Seven miRNA (bold) are downregulated at least 2-fold in TRAMP prostate as compared to wild type at either 16 or 21 week old animals. Fold change is calculated as TRAMP expression as compared wild type at each time point. Expression normalized to U6. N = 3 animals per time point per genotype.

* p < 0.05;

** p < 0.01;

*** p < 0.001.

**Figure 3:**
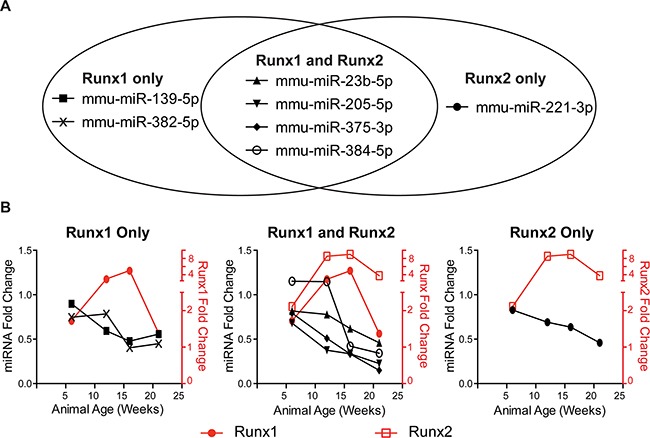
Runx-targeting miRNAs have reciprocal expression trends to Runx factors during PCa progression Of the 28 miRNAs screened in Table 1 in (**A**), seven miRNAs predicted to target Runx1, Runx2, or both are downregulated in TRAMP prostates as compared to age-matched, non-transgenic control animals. In (**B**), fold change is calculated as TRAMP expression compared wild type at each time point. mRNA expression normalized to 18S and miRNA expression normalized to U6. N = 3 animals per time per genotype. Error bars are not shown for simplicity.

Using the TRAMP mouse, we identify clinically relevant miRNAs targeting the Runx family (Figure 3). Several of these candidate miRNAs, such as miR-23b-5p [51] and miR-221-3p [52], have a characterized phenotype in PCa and are downregulated in malignant human tissue [53]. Four miRNAs target both Runx1 and Runx2 and are sharply downregulated by 12 weeks coincident with the peak levels of Runx factors (Table 1-bold and italic, Figure 3). These are miR-23b-5p and miR-205 which function as tumor suppressors in many ways [26, 51], miR-375-3p is well characterized as a valuable marker of disease progression for diagnosis and prognosis [reviewed in 54], and miR-384-5p. Currently, for miR-384-5p, there is no information related to cancer and therefore represents a potentially novel function in PCa. Similar downregulated expression patterns are observed for candidate miRNAs predicted to regulate either Runx1 or Runx2 (Table 1-bold and italic, Figure 3). Runx1 is uniquely targeted by miR-139-5p and miR-382-5p, neither miRNA has been extensively examined in association with prostate cancer. One study showed that miR-139-5p secreted from primary prostate cancer cell cultures increased the expression of genes that can contribute to the invasive properties of PCa cell populations [55]. miR-382-5p has been reported to inhibit tumor growth and enhance chemosensitivity in osteosarcomas [56]. miR-221-3p, an established PCa tumor suppressor [52], uniquely targets Runx2. Together these miRNAs suggest complex and potentially redundant mechanisms to prevent dysregulation of Runx factors in the healthy prostate.

### A Runx/miRNA interaction network drives prostate cancer

**Figure 4:**
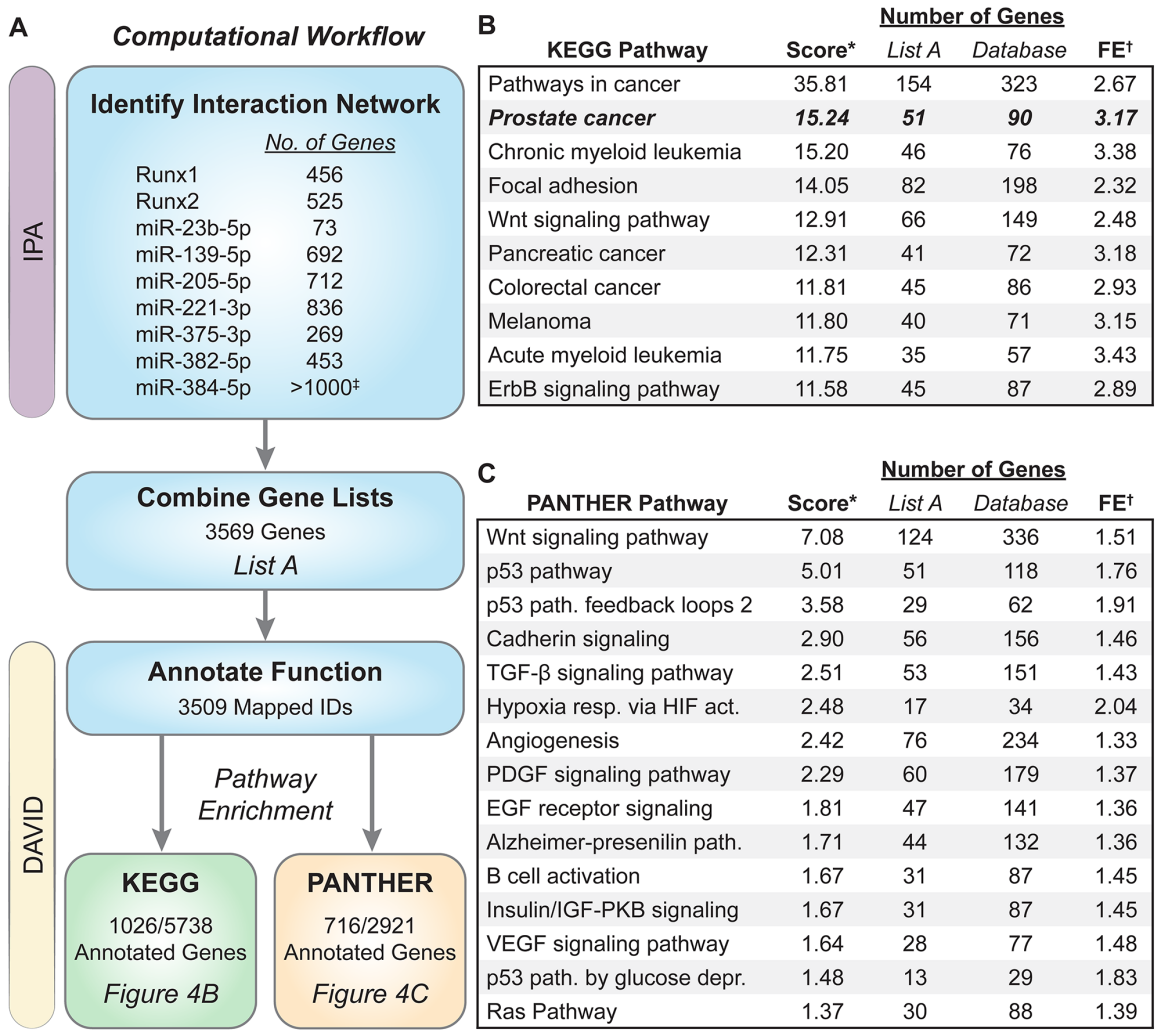
Biological pathway analysis of genes in the Runx/miRNA interaction network The computational workflow used to discover enriched pathways is outlined in (**A**) Genes that interact directly or indirectly with Runx1, Runx2, or the seven candidate miRNAs were identified in IPA and exported. These nine lists were combined into *List A* containing 3569 genes. *List A* genes were upload in DAVID v6.7, annotated for function, and databases queried to identify pathway enrichment ranked by EASE score (modified Fisher Exact test). *List A* contains ~18% of the genes within the KEGG database and ~25% contained in the PANTHER database. The top 10 KEGG pathways are shown in (**B**) and 16 PANTHER pathways with an EASE score < 0.05 shown in (**C**) *Score is the −log_10_(EASE score). ^†^FE is fold enrichment within *List A* as compared to all annotated genes within each database. ^‡^miR-384-5p has over 1000 gene interactions in IPA. Only the top 1000 were used in our analysis.

A combined Runx/miRNA regulatory network that promotes prostate carcinogenesis has not been previously described. To discover the molecular mechanisms through which Runx1, Runx2, and the Runx-targeting miRNAs, miR-23b-5p, miR-139-5p, miR-205-5p, miR-221-3p, miR-375-3p, miR-382-5p, and miR-384-5p, drive prostate tumorigenesis, we interrogated well-accepted bioinformatics tools; DAVID [57, 58] and Ingenuity Pathway Analysis (IPA-www.ingenuity.com). Genes that interact with Runx1 (456 genes), Runx2 (525 genes), or the seven candidate miRNAs (ranging from 73 to >1000 targets) were identified in IPA. These genes were combined into a master list, *List A* (Supplementary Table S1), of Runx/miRNA (3569 genes) interacting factors. *List A* genes were imported into DAVID and annotated for function across numerous database. Enriched ontologies and biological pathways within the KEGG and PANTHER databases were determined and ranked by EASE score (Figure 4A). Seven of the top 10 enriched KEGG pathways are related to a specific cancer with *Prostate cancer* being the second most significant pathway with an over 3-fold enrichment as compared to all annotated genes (Figure 4B). Moreover, interrogation of the PANTHER database identifies cancer (p53 and Ras) and bone (Wnt and TGF-β) related pathways, as well as many growth factors (PDGF, EGF, IGF, and VEGF), to be significantly enriched within the Runx/miRNA interaction network (Figure 4C).

**Figure 5:**
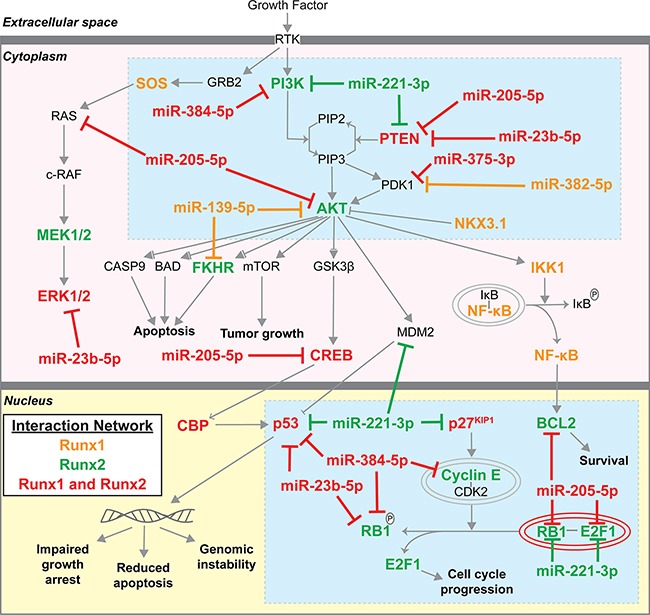
Runx1/2 and targeting miRNA molecular interactions in *Prostate Cancer Signaling* network Genes that interact with Runx1, Runx2, and selected Runx targeting miRNAs (Figures 3, 4) were identified using IPA (www.ingenuity.com) and mapped to the *Prostate Cancer Signaling* network. Orange genes interact with Runx1; Green genes interact with Runx2; Red genes interact with both Runx1 and Runx2. Blue boxes highlight PI3K/AKT/PTEN and control of cell growth as centers through which Runx1, Runx2, and Runx-targeting miRNAs function.

We next mapped the genes in the interaction networks of Runx1, Runx2, coupled or linked to the seven candidate miRNAs (see Table 1 bolded miRNAs) onto the *Prostate Cancer Signaling* (PCS) network within IPA (Figure 5). In addition to targeting Runx1 and Runx2, our candidate miRNAs regulate numerous other genes within the PCS network. While both Runx1 and Runx2 have unique interaction partners, orange and green highlighted genes respectively, many genes and miRNAs interact with both Runx1 and Runx2, highlighted in red. Two focal points are revealed, centered on PI3K/AKT/PTEN and cell growth control (p53/p27^Kip1^), as highlighted by blue boxes, suggesting potential mechanisms of action through which Runx factors and targeting miRNAs regulate prostate cancer. Upon query of data generated by the TCGA Research Network, AKT and PI3K tend towards co-occurancy with Runx1 and Runx2, whereas PTEN tends towards mutual exclusion [15, 17]. The latter is consistent with the observed upregulation of Runx1 and Runx2 when PTEN is downregulated by 21 weeks in the diseased TRAMP prostate (Figure 1A, 1B, 1G), while the former supports the proposed idea that mutual activation of Runx and PI3K/AKT signaling promotes tumor progression [2].

## DISCUSSION

The goal of our study is to elucidate mechanisms underlying the deregulated expression of Runx factors during PCa progression. Here, we identify miRNAs targeting Runx factors with reciprocal changes that reflect abnormal Runx expression levels at stages of advancing tumorigenesis in the TRAMP mouse. Our study, for the first time, identifies changes in Runx1 and Runx2 that are associated with molecular markers of human prostate cancer progression. The significant findings of our study relating TRAMP mouse to human prostate cancer include: 1) a transition stage from androgen-sensitive to androgen-insensitive disease as indicated by loss of PTEN and the androgen receptor; 2) constitutively elevated Runx1 and Runx2 in PCa with reciprocal downregulation of seven Runx-targeting miRNAs, as compared to wild type; 3) functionally, both Runx1 and Runx2, when downregulated, reduce cell motility; 4) miRNAs targeting Runx1 and Runx2 also target genes linked to other PCa associated pathways, including Wnt, TGF-β, p53, IGF, EGF, VEGF that are all known to be regulated by Runx factors [13, 59]; and 5) the interaction network based on Runx1, Runx2, and Runx-targeting miRNAs is centered on PI3K/AKT signaling.

The funneling of downregulated miRNAs targeting Runx factors and other gene targets revealed in the *Prostate Cancer Signaling* network identify molecular interactions with key signaling pathways promoting tumor growth similar to human PCa progression (Figure 5, e.g., PI3K, AKT, ERK, p53, RB/E2F). Specific and coordinated activities of Runx1 and Runx2 are identified during prostate tumorigenesis. The presence of miRNAs targeting Runx1 and/or Runx2 in normal prostate tissue and the complete absence of these miRNAs in prostate tumors upregulates both Runx1 and Runx2 as well as AKT signaling. Progression through AKT signaling in highly relevant to human PCa. Phosphorylation of Runx2 increases Runx2 transcriptional activity [2], and occurs in response to most growth factor signaling as confirmed in PCa by pathway analysis (see Figure 4C).

The impact of our study not only elucidates the involvement of Runx1 and Runx2 during tumorigenesis, but also identifies stages of disease progression at the molecular level within the TRAMP model system. The initial upregulation of both Runx1 and Runx2 that we describe here, parallels the increase in AR previously reported in androgen-dependent human PCa [60, 61]. Most significant to human PCa is the change that we identify in the TRAMP mouse, for the first time that occurs between 16 and 21 weeks as the disease transitions from AR positive to AR negative tumors. In Table 1, numerous miRNAs tied to androgen response in PCa are strikingly downregulated, such as miR-27b-3p, miR-141-3p, miR-181a-5p, miR-221-3p, and miR-375-3p. Consistent with these observations is the fact that miRNAs predicted to target AR, such as miR-23b-5p, miR-181-5p, and miR-205-5p are constitutively reduced in TRAMP prostates. Importantly, these miRNAs also target Runx factors. Thus, the inflection point of disease progression between 16 and 21 weeks marked by the loss of AR is coupled to further de-repression of Runx1 and Runx2. The combined effects of loss of miRNAs in prostate tumors that regulate AR and Runx in normal prostate tissue, contribute to tumorigenesis in the TRAMP mouse, mirroring that of human disease. Given these findings, the TRAMP mouse can be used to pursue the mechanisms contributing to this dramatic change in PCa markers to enhance our understanding of disease progression. Moreover, Runx-targeting miRNAs inform a regulatory network that translates to an improved understanding of prostate biology and novel options for prostate cancer diagnosis and directed therapy.

## MATERIALS AND METHODS

### Ethics statement

Investigation has been conducted in accordance with the ethical standards and according to the Declaration of Helsinki and according to national and international guidelines and has been approved by the authors' institutional review board. In conducting research using animals, the investigators adhere to the laws of the United States and regulations of the Department of Agriculture.

### Mice

Hemizygous females C57BL/6-Tg(TRAMP) 8247Ng/J (strain 003135, The Jackson Laboratory) were crossed with wild type C57BL/6 males. All animals were genotyped using the following PCR primers: Transgene oIMR7084: 5′-GCGCTGCTGACTTTCTAAACATAA G-3′; Transgene oIMR7085: 5′-GAGCTCACGTTAAGTTTTGATGTGT-3′; Int. pos. CTL F oIMR7338: 5′-CTAGGCCACAGAATTGAAAGATCT-3′; Int. pos. CTL R oIMR7339: 5′-GTAGGTGGAAATTCTAGCATCATCC-3′. All animals were housed in a pathogen-free environment and handled according to protocol number 12-054 approved by the Institutional Animal Care and Use Committee at the University of Vermont.

### Dissection of ventral and lateral murine prostate lobes

Prostate lobes were removed as previously described [62].

### RNA isolation

Prostate lobes were washed in 1X PBS and transferred to 1 mL QIAzol (QIAGEN) for tissue homogenization. Tissue was homogenized on ice in three to six 5-second bursts with a Polytron 2100 homogenizer with a 7mm tip on setting 25. Homogenized tissue in QIAzol was stored at −80°C until RNA isolated. RNA was isolated with the miRNeasy Mini kit (QIAGEN) following manufacture's protocol. RNA quality was assessed on an Agilent 2100 Bioanalyzer using RNA Nano chips based on the RIN number, ratio of 28S/18S, and presence of distinct of 18S and 28S peaks with lack of RNA fragments less than 1000 nucleotides.

### Quantitative RT-PCR

cDNA for miRNA and mRNA was prepared separately using 1000ng of RNA. mRNA cDNA was synthesized with the SuperScript III First-Strand Kit (Life Technologies) and miRNA cDNA was synthesized with the miScript II RT Kit (QIAGEN) using the HiSpec buffer following the recommended manufacturer's protocol. All qPCR was performed in 384-well plates on an ABI ViiA7 machine using iTaq Universal SYBR Green (Bio-Rad) or QuantiTect SYBR Green (QIAGEN) for mRNA and miRNA respectively. Data was normalized to expression of 18S and U6. Forward miRNA primer and forward and reverse mRNA primer sequences are listed in Supplementary Table S2.

### Cell culture and transfection

TRAMP-C2 cells, an epithelial line isolated from tumor burdened TRAMP prostates, were maintained in High-Glucose DMEM (Hyclone) supplemented with 0.005 mg/ml bovine insulin (Sigma), 10nM dehydroisoandrosterone (Sigma), 5% fetal bovine serum (Atlanta), 5% Nu-Serum IV (Corning), and 1% penicillin/streptomycin (Life Technologies). One day prior to transfection, cells were plated at 4 x 10^5^ cells/well in 6-wells plates. 50nM Dharmacon ON-TARGETplus SMARTpools for non-targeting control #1 (D-001810-01-05), siRunx1 (L-048982-00), or siRunx2 (L-012665-00-0005) were transfected into cells with Lipofectamine 2000 (Life Technologies) as per manufacturers recommended protocol.

### Migration assay

Following transfection, cells were incubated overnight in reduced serum media (1% FBS and 1% Nu-Serum IV). A scratch along the diameter of each well was made in the cell monolayer with a 200μL pipet tip and sufficiently washed to remove floating debris. Three pre-marked locations were imaged along each scratch at four-hour intervals for 20 hours. Images were acquired in SPOT Imaging Solutions Software v5.0 from a SPOT RT3 2Mp Monochrome camera (Diagnostic Instruments, Inc.) attached to an Eclipse TS100 (Nikon) microscope using a 10X/0.30 Plan Fluor objective (Nikon). Scratch area was measured with the MiToBo plug-in for ImageJ (Fiji) using default settings.

### Bioinformatics

Candidate miRNAs targeting Runx1 and Runx2 were identified using TargetScan v6.2 (www.targetscan.com). IPA (www.ingenuity.com QIAGEN) was used to identify molecular interaction networks of Runx1, Runx2, and selected miRNAs. Gene lists were compiled into one dataset and analyzed with DAVID v6.7 (https://david.ncifcrf.gov/) using default settings.

### Statistics

All data were graphed and analyzed using Prism v6 (GraphPad Software) to calculate SEM and p-values (unpaired t-test).

## SUPPLEMENTARY FIGURE AND TABLES






