# Supplementary material for: A microRNA/Runx1/Runx2 network regulates prostate tumor progression from onset to adenocarcinoma in TRAMP mice
Source: Oncotarget. 2016 Sep 13;7(43):70462–74. doi: 10.18632/oncotarget.11992 (PMC5342565; doi:10.18632/oncotarget.11992)
Supplement: Supplementary file 1 [file oncotarget-07-70462-s001.pdf]

A microRNA/Runx1/Runx2 network regulates prostate tumor progression from onset to adenocarcinoma in TRAMP mice

SUPPLEMENTARY FIGURE AND TABLES

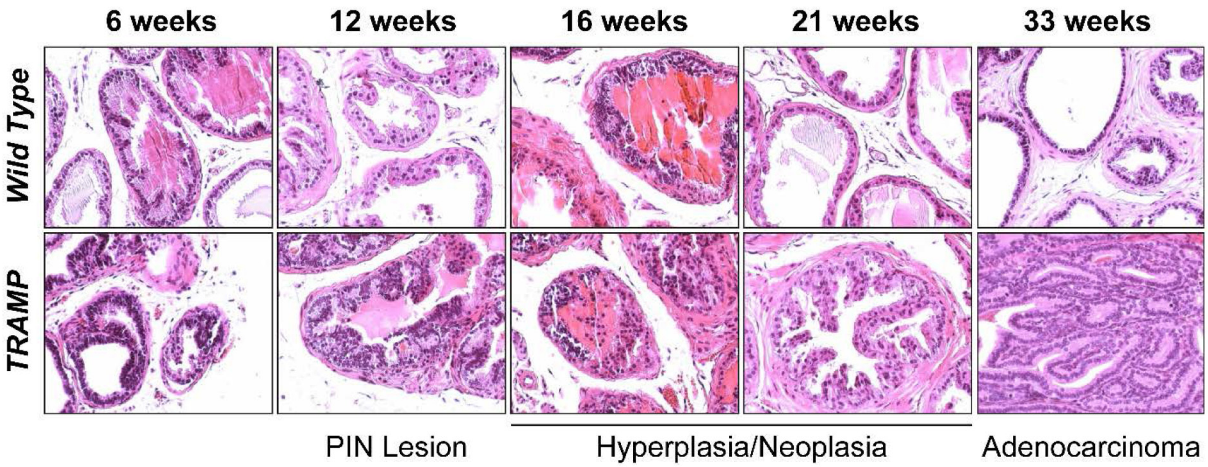

Supplementary Figure S1: Histology of TRAMP prostate lobes from PIN lesion through adenocarcinoma. Representative tissue sections of TRAMP or wild type prostate lobes stained for hematoxylin (pink) and eosin (purple) from six weeks through 33 weeks show progression of disease states (as indicated) from normal glandular tissue to dedifferentiated, adenocarcinoma in TRAMP animals.

Supplementary Table S1: Gene *List A* as indicated in Figure 4.

See Supplementary File 1

Supplementary Table S2: qRT-PCR primer sequences.

See Supplementary File 2
